# Does Human Capital Matter for China’s Green Growth?—Examination Based on Econometric Model and Machine Learning Methods

**DOI:** 10.3390/ijerph191811347

**Published:** 2022-09-09

**Authors:** Xiaoxue Liu, Fuzhen Cao, Shuangshuang Fan

**Affiliations:** 1 School of Economics, Beijing Technology and Business University, Beijing 100048, China; 2 School of Management, China University of Mining and Technology-Beijing, Beijing 100086, China

**Keywords:** human capital, green economy efficiency, green innovation, LightGBM machine learning, green growth, industrial upgrading

## Abstract

To tackle the increasingly severe environmental challenges, including climate change, we should pay more attention to green growth (GG), a path to realize sustainability. Human capital (HC) has been considered a crucial driving factor for developing countries to move towards GG, but the impact and mechanisms for emerging economies to achieve GG need to be further discussed. To bridge this gap, this paper investigates the relation between HC and GG in theory and demonstration perspective. It constructs a systematic theoretical framework for their relationship. Then, it uses a data envelopment analysis (DEA) model based on the non-radial direction distance function (NDDF) to measure the GG performance of China’s 281 prefecture level cities from 2011 to 2019. Ultimately, it empirically tests the hypothesis by using econometric model and LightGBM machine learning (ML) algorithm. The empirical results indicate that: (1) There is a U-shaped relationship between China’s HC and GG. Green innovation and industrial upgrading are transmission channels in the process of HC affecting GG. (2) Given other factors affecting GG, HC and economic growth contribute equally to GG (17%), second only to city size (21%). (3) China’s HC’s impact on GG is regionally imbalanced and has city size heterogeneity.

## 1. Introduction

The global industrialization and urbanization have disturbed the earth’s natural balance. The critical imbalance in the carbon cycle between carbon sources and carbon sinks has forced the world to focus on issues of global warming and frequent natural disasters. The increasingly severe climate change has significantly impacted ecosystems and economics, as well as social development [1,2,3]. As the world’s leading developing economy, China has become the world’s largest carbon emitter [4] in recent years. Therefore, it is China’s duty as a major power to transfer its development model to reduce energy consumption and carbon emissions while maintaining economic growth. To this end, at the 2015 Paris Climate Conference, the Chinese government made a commitment to hitting peak carbon emissions by 2030 [5]. Since then, the green growth model based on harmonious coexistence of humans and nature has become the core value orientation in China.

The concept of green growth was first proposed in the United Nations Economic and Social Commission for Asia and the Pacific (UNESCAP) in 2005. Green growth emphasizes that, when reducing poverty and improving human well-being through economic growth, countries should focus on transforming economic growth and consumption patterns, improving the ecological efficiency of economic growth, and coordinating environmental and economic development [6], so as to achieve sustainable development goals. The concept of green growth takes green economy, low-carbon economy, and circular economy as the main economic forms [7]. Its primary goal is to promote the transformation and upgrading of industrial civilization to ecological civilization [8]. However, the transition from brown economy to green economy will involve government policies [9,10], economic growth target pressure [11,12], technological progress [13], socio-cultural contexts [14], transition costs [15,16,17], and many other factors. Among many potential factors affecting green growth, human capital, known as a kind of living capital, refers to the knowledge and skill sets that workers have [18,19,20]. It is characterized by creativity, innovation, and subjective initiative, so it contributes greatly to green growth patterns [21,22].

Since China’s reform and opening up, the human capital level has continued to improve [23]. According to the *China Human Capital Report 2021* (http://news.cufe.edu.cn/info/1002/52212.htm, accessed on 14 December 2021) released by China Center for Human Capital and Labor Market Research of Central University of Finance and Economics, the average education years for China’s labour force increased from 6.1 years in 1985 to 10.5 years in 2019. The national workforce population with a college degree or above increased from 10% to 20.6%. This naturally arouses a series of questions worthy of attention: what is the relationship between China’s human capital and green growth? What is the internal mechanism driving the formation of such relationship? What is the transmission path? Among many factors influencing green growth, how much does human capital contribute? Although academics and policy makers pay more and more attention to these issues, few of them can provide theoretical and empirical findings that systematically answer those questions.

Research conclusions in existing literature on the relationship between human capital and green growth are inconsistent. A lot of literature believes that there is a linear relationship between human capital and green growth. They propose two kinds of distinct conclusions: one is “promotion viewpoint”, indicating that human capital promotes green growth (or reduces carbon emissions) [24,25,26,27,28,29]; the other is “inhibition viewpoint”, arguing that human capital inhibits green growth (or increases carbon emissions) [30,31]. Other literature believes that the relationship between human capital and green growth is uncertain, changing with different time periods, different industries, macroeconomic variables and human capital regime level in various regions [32,33,34]. A few studies confirm the possible non-linear relationship between human capital and green growth [35,36,37]. In existing studies on the nonlinear relationship, both the measurement index and nonlinear shape of green growth and human capital are quite different. Reviewing conclusions about human capital and green growth, such as the promotion viewpoint, inhibition viewpoint and non-linear relationship viewpoint, we find it necessary to further clarify the relationship between human capital and green growth. Specifically, for emerging economies such as China, it will help them promote green growth more efficiently.

To answer the previous questions, by using the panel data of China’s 281 prefecture-level cities (including municipalities directly under the Central Government) from 2011 to 2019, this paper examines the relationship between China’s human capital and green growth from theoretical and empirical perspectives. Hence, we can precisely classify and implement specific policies. First, from a theoretical perspective, we propose the hypotheses that there is the nonlinear relationship between human capital and green growth, and that green innovation and industrial upgrading are transmission paths. Meanwhile, in order to reveal the time series trend and spatial distribution characteristics of China’s green growth, this paper uses the data envelopment analysis (DEA, Appendix A) model based on the non-radial direction distance function (NDDF) to measure the sample cities’ green economic efficiency (GEE). Furthermore, this paper empirically tests the previous hypotheses. Specifically, it uses econometric models to investigate whether human capital and green growth have the nonlinear relationship and the main transmission path. It also applies machine learning algorithms to measure the human capital’s contribution weight among many influencing factors. The research findings are as follows: first, China’s human capital and green growth have a U-shaped relationship rather than a simple linear relationship. That is, when human capital development cannot reach a certain threshold, it will inhibit green growth; when it exceeds a certain threshold, it will promote green growth. This conclusion is still reliable after the robustness test. Green innovation and industrial upgrading are transmission channels in the process of human capital affecting green growth. Second, the result of the machine learning algorithm reveals that among many factors influencing green growth, the human capital’s contribution weight is higher, at about 17%. It is as important as the economic growth level, second only to city size (21%). In addition, the heterogeneity analysis indicates that human capital has exceeded the U-shaped threshold in southern regions. In the eastern region, it has been near the U-shaped threshold and is about to promote green growth. In other regions, human capital has not yet been able to promote green growth. In large cities, human capital has already exceeded the U-shaped threshold. While in small and medium-sized cities, it is still on the left side of the U-shaped threshold, indicating that city size can speed up crossing a threshold between human capital and green growth so that human capital can positively promote green growth.

This study provides the following three contributions: first, it illustrates the theoretical root of the nonlinear relationship between human capital and green growth from the production and consumption perspective. Meanwhile, the inner mechanism of human capital influencing green growth is analyzed in detail. It proposes two transmission paths of green innovation and industrial upgrading. This work directly proves that there is a U-shaped relationship between human capital and green growth, which enriches and expands the research results of nonlinear relations between them [35,36,37]. This means that the linear relationship assumptions between human capital and green growth, i.e., the promotion or inhibition viewpoints, are not suitable for China. Second, this study uses an econometric model and machine learning (ML) algorithms to test theoretical hypotheses, which not only clarifies the transmission mechanism of human capital affecting green growth, but also introduces advanced ML algorithms into economics field to study human capital’s contribution to green growth. However, existing studies on nonlinear relationship [35,36,37] only use econometric models for empirical research, which cannot accurately reflect human capital’s contribution to green growth. Third, this study conducts a series of grouping heterogeneity tests based on the city’s location and size, respectively, and uses the U test econometric model to examine whether the human capital development level in different groups exceeds the U-shaped threshold. Hence, we can adjust measures to local conditions and implement the classified policies to ensure that human capital will positively impact green growth policy.

The remainder of this paper is organized as follows. Section 2 provides theoretical basis and research hypothesis. Section 3 discusses research design and data selection. Section 4 reveals empirical results of econometric models and machine learning algorithms. Section 5 concludes by proposing main conclusions and policy implications.

## 2. Literature Review and Hypothesis Proposal

### 2.1. Human Capital and Green Growth

Human capital refers to labour’s ability composed of knowledge, skills, and physical ability. It is formed through human investment (such as education investment), takes workers as a carrier, and indicates worker’s skills, intelligence and talents [18,20]. Schultz, the “father of human capital theory”, believes that human capital is the source of driving economic growth. He also emphasized the important role played by the “quality” of human capital [38]. In the green transformation of the economy, human capital also contributes important value. Recently, some scholars have studied the relationship between human capital and green economy, but their conclusions are inconsistent. These conclusions include “promotion viewpoint”, “inhibition viewpoint” and “non-linear relationship”. The “promotion viewpoint” holds that human capital can improve natural resource conservation [24,28,39], reduce energy consumption intensity [25], and reduce pollutant emission [26,27,29]. The “inhibition viewpoint”, on the contrary, emphasizes the positive correlation between human capital and carbon emissions [30,31]. Based on “promotion viewpoint” and “inhibition viewpoint”, some scholars argue that the relationship between human capital and carbon emissions is time-varying. It varies in the short-term and long-term, in different industries as well as financial development and human capital at different regime levels [2,32,33]. Li and Ou Yang [33] argue that human capital increases CO_2_ emissions in the short term and reduces CO_2_ emissions in the long term. Çakar et al. [34] find that financial development and the development level of human capital affect whether human capital increases or suppresses carbon emissions. Human capital increases carbon emissions in both low regimes of financial development and human capital, and decreases in high regimes. In addition, some studies believe that human capital has a significant threshold effect on the green economy, resulting in nonlinear effect under different levels of complex variables of economic and social development [35,36,37]. Liu and Lv [36] test the non-linear relationship between rural human capital and agricultural green total factor productivity (AGTFP) in China. Maranzano et al. [37] test the nonlinear relationship between education and emissions, reflecting the dynamic change in OECD and European economic and social development. Chen et al. [35] believe that green R&D activities and sulfur dioxide emissions are in a nonlinear relationship, but are affected by technology absorption capacity. At present, the view that human capital and green growth have a nonlinear relationship comes from indirect evidence rather than direct evidence. There are great differences in the measurement of human capital and green growth. The existing green growth indicators include AGTFP, CO_2_ emissions, SO_2_ emissions and other measurement indicators. Human capital includes rural human capital, average years of education (population 15–64 years), green R&D and other measurement indicators; furthermore, the nonlinear shape is also inconsistent, and it is considered as an “N-shaped” relationship [36] or an inverted U-shaped relationship [37]. However, the human capital formed through education investment needs to be accumulated for a relatively long time before population endowment improves [40], which in turn positively affects the green growth. Therefore, we believe that human capital and green growth are not a simple linear relationship, but have different impacts on the green economy at different human capital development stages.

First, from the production sector perspective, human capital is closely related to productivity [41,42]. When human capital is at a low development level and employees have low education level and professional skills, the industry will absorb a large number of low-skilled labours and have very few high-skilled labourers [43]. Under such circumstances, the marginal contribution rate of talents to production is low, and the output improvement mainly depends on the large-scale investment of physical capital, which leads to “high energy consumption and high pollution emissions” that hinder the green economy. As human capital continues to accumulate and enters a higher development stage, the labour skill structure changes; the proportion of high-skilled labour increases significantly, and the complementarity between capital and skills begins to strengthen [44]. Hence, the individual production department’s efficiency is significantly improved at first, and generates a positive spillover effect through the demonstration effect [45], driving the entire production department to reshape the production process to reduce physical capital input and improve production through technological iteration. It then further reduces energy consumption, pollution levels, and promotes green economy development. Second, from the consumption perspective, the human capital level is closely related to the consumption structure [46,47]. The low-level human capital development stage corresponds to the relatively low consumers’ income and affordability [48]. Under such circumstances, as human capital improves, consumers often pay attention to related consumer goods to meet basic “material needs”, such as purchasing household appliances, automobiles, and other large commodities. However, if such consumer demand continues to grow, it will increase carbon dioxide emissions and inhibit green growth [49]. As human capital development exceeds a certain level, on the one hand, after the basic “material needs” are fully satisfied, the consumption structure will undergo a “qualitative leap”; that is, “spiritual consumer goods” related to entertainment and health will take the lead. On the other hand, high-level human capital with good environmental awareness [50] will increase the consumption proportion of environmentally friendly, green, and low-pollution consumer goods that are conducive to green growth. In addition, consumption structure upgrade will also force the production sector to improve and iterate products [6,49,51], which is conducive to producing more environmentally friendly products [52]. Therefore, human capital at this stage will positively promote green economy development.

To sum up, the relationship between human capital and green growth is not a simple linear one. If the human capital does not reach a certain threshold, it will inhibit green growth. On the contrary, it will promote green growth. The human capital will first inhibit green growth and then promote it. Accordingly, we put forward the following hypothesis.

**Hypothesis** **1.** *There is a U-shaped relationship between human capital and green growth*.

### 2.2. Human Capital, Green Innovation and Green Economic Efficiency

Romer’s endogenous growth theory believes that human capital is an important source of driving total factor productivity improvement and technological progress. Human capital promotes innovation from both micro and macro perspectives. At the micro level, human capital represents a high level of human resources and can directly affect R&D activities within a company [53,54]. High-level human resources can promote a company’s technology R&D through integrating both internal knowledge and external knowledge [21,55]. This integration mainly includes knowledge creation, knowledge dissemination, knowledge diffusion, and companies’ internal R&D activities transformation [56]. Therefore, high-level human capital can directly affect companies’ R&D activities, thereby enhancing their innovation level. On the other hand, at a macro level, when a city’s human capital is at a high level, it can bring about knowledge spillover effect through the agglomeration, flow, and imitation of talents [57,58]. That is, companies with high-level human capital can share and transfer their tacit knowledge and resources to other companies in the industry chain to drive the entire industry chain and city to innovate and develop [59,60]. In short, human capital will promote innovation and development.

However, existing studies have shown that the relationship between green innovation and GEE is often nonlinear. Chen and Huo [61] and Shi et al. [62] argue that there is an inverted U-shaped relationship between innovation and carbon emissions. Hu et al. [63] finds that there is a U-shaped relationship between green innovation and green development. First, corporate innovation requires enterprises to expand their investment through years of operation and accumulation. In the process of realizing its technological innovation, enterprises will spare no effort to increase R&D investment in the early stage [64]. However, due to the long cycle and high risk of scientific and technological innovation, early R&D investment may not be able to be converted into R&D results in time to play the role of driving the city’s green growth [35]. Moreover, since now company scale expansion and capital recycling have brought certain negative externality to the environmental system, the “rebound effect” of such negative externality is greater than the energy-saving effect brought by technological innovation [65,66], which will increase energy consumption and carbon emissions to some extent, and is not conducive to improving green economic performance [61,62]. Second, when the innovation level exceeds a certain threshold and reaches a high level, the early R&D investment is transformed into a real force to promote companies’ technological improvement and product iteration. Hence, the innovation results can be transformed and resource use efficiency is improved, thus reducing carbon emissions [67,68] and achieving high-level green development [63,69]. Therefore, we propose the second hypothesis.

**Hypothesis** **2.** *Green innovation is the intermediate variable between human capital and green growth*.

### 2.3. Human Capital, Industrial Upgrading, and Green Growth

British economist Crick was the first to interpret the connotation of industrial upgrading. That is, when the labour force transfers from the primary industry to the secondary and tertiary industries, a country’s economy gradually evolves from the primary industry as the leading industry to the secondary and tertiary industries [70]. During industrial upgrading process, labour, capital, natural endowment, and technological progress have become important driving factors [71,72,73]. As a “living capital”, human capital, with labour as its carrier, has greater value-added potential than hard capital such as capital and material, and is more innovative and creative [21,53]. Therefore, it has a non-negligible contribution rate to industrial upgrading [72,74,75]. Schultz believes that education is the most important form of human capital investment [76]. The improvement of labour education level has accelerated its transfer from the primary industry to the secondary and tertiary industries [73]. The accumulation of human capital stock also will help to break the original industrial chain and accelerating the process of forming a new economic and technological industrial chain, which will lead to changes in the industrial and market environment and help to create a new industrial chain [77,78]. In addition, the higher the level of human capital stock, the stronger the efficiency of knowledge dissemination and spillover, that is, the better the effect of “learning by doing”, which is conducive to transforming and absorbing advanced technology, thus boosting the industrial structure leap [79].

However, the industrial upgrading process requires a leap from the accumulation of quantitative changes to qualitative changes, which is not achieved overnight. The relationship between industrial upgrading and green growth is not a simple linear one. The “accumulation” stage and “leap” stage of industrial upgrading may have different impacts on green growth. Existing studies, such as Wei and Zhang [80], Liang et al. [81], Yang et al. [82], and Zhang et al. [83], demonstrate the nonlinear relationship between the two. In the initial stage of industrial upgrading, since a large amount of capital and labour flow into the secondary and tertiary industries, and market-driven industrial changes often lack scientific policy supporting facilities [84], this type of industrial upgrading is relatively extensive. The profit-seeking nature of capital makes the industry focus on the return on investment measured in currency, while ignoring the governance of externalities such as environmental pollution [85,86]. As the industrial upgrading reaches a certain level, the industry gradually transforms from a low value-added, extensive, low-tech one to a high value-added, intensive, and high-tech one [87,88]. Meanwhile, with the implementation of a series of high-quality development strategies, innovation-driven, green development, and other initiatives drive the industrial upgrading process and the green development to run simultaneously, which actively promotes the GEE [89,90,91]. Based on this, we propose the third hypothesis:

**Hypothesis** **3.** *Industrial upgrading is the intermediate variable between human capital and green growth*.

## 3. Methodology and Data

First, this section explains the variables selected in this study. Second, we apply the NDDF-DEA model to measure cities’ green growth level during the statistical period. Then, we employ the econometric models and LightGBM machine learning model to explore the impact and mechanism of human capital on green growth in this study. Finally, the data source used in this study is briefly introduced.

### 3.1. Variable Measurement and Selection

The variables selected in this study are shown in Table 1.

#### 3.1.1. Dependent Variable: Green Economic Efficiency (GEE)

Green growth is the dependent variable in this study. Referring to Cheng et al. [92], as well as Wang and Chen [93], this study uses green economic efficiency (*GEE*) as the proxy variable of green growth. This study originally uses distance functions [94], including the Shephard distance function (SDF) and directional distance function (DDF), when measuring *GEE*. However, SDF cannot achieve pollutant emission reduction when ensuring an ideal output [94]. Although DDF overcomes this problem, it leads to an overestimation of efficiency [95]. On this basis, Zhou et al. [96] proposed NDDF.

This study introduces the DEA model to measure the *GEE* of sample cities. This model has the advantage of comprehensively considering the desirable outputs and undesirable outputs in the economic system from the aspects of input and output. In addition, Zhang and Li [97] and Li and Ji [98] both use NDDF of the DEA model to measure *GEE*. The input variables include energy (E), labour, and capital. In terms of output variables, the desirable output is GDP, and the undesirable outputs are industrial wastewater (WW), industrial sulfur dioxide gas (WG), and industrial soot and dust (SD), as well as carbon dioxide (CD). In this process, the weights of the energy input (E), GDP, WW, WG, and SD are set to 1/3, 1/3, 1/9, 1/9, and 1/9, respectively. The proportion of these five weights, which can be increased or decreased, is calculated by the super-efficiency DEA model. Finally, the GEE of the *i*-th city in the *t*-th period is constructed as the dependent variable of this article.

$$DDF_{it}=\frac{1}{2}\left[\frac{\left(E_{it}-\beta_{E,it}*E_{it}\right)/\left(G_{it}-\beta_{G,it}*G_{it}\right)}{E_{it}/G_{it}}\right]+\frac{1}{2}\left[\frac{1}{3}\sum_{N=WW,WG,SD}\frac{\left(N_{it}-\beta_{N,it}*N_{it}\right)/\left(G_{it}-\beta_{G,it}*G_{it}\right)}{N_{it}/G_{it}}\right]$$

where *β_E_*, *β_G_*, *β_WW_*, *β_wG_*, *β_SD_* are the optimal solutions of the DEA model. Capital stock data are calculated using the perpetual inventory method. The raw data required include the fixed asset investments of cities at the prefecture level and above; these data are obtained from the CEIC China Economy Database. Based on the estimation reported by Xiang [99], we can obtain the capital stock data of each city in the base year (2000) and the capital stock depreciation rate of each city [99]. We agree that the capital stock depreciation rates of cities in the same province are the same.

#### 3.1.2. Independent Variable and Intermediate Variables

(1) Core Independent Variable: Measurement of Human Capital

The core variable considered in this article is the human capital. Human capital has many measurement dimensions, but mainly focuses on education [22,100]. Existing studies include the number of college students [22] and the average years of education of the population [100,101]. In addition, some studies have pointed out that government education expenditure is highly correlated with human capital formation and development [101,102]. Therefore, this paper uses the logarithm of urban government education expenditure to measure city human capital level. It also uses the average education years of the population [22] as the proxy variable of human capital for robustness test.

(2) Intermediate Variable: Green Innovation (GIN)

High-level human capital promotes green innovation [59]. Green innovation (*GIN*) will largely affect carbon emissions [61], affecting green economic development [63]. In this paper, green innovation is measured by the number of green invention patent applications in prefecture level cities.

(3) Intermediate Variable: Industrial Upgrading

Existing literature reveals that human capital will lead industrial upgrading [73]. Industrial structure in turn will certainly impact energy efficiency [103,104]. Industrial upgrading is now the main form of industrial structure change. Referring to Yao et al., (2019) [105], this study uses the ratio of the secondary and tertiary industries’ total output value to GDP to measure industrial upgrading.

#### 3.1.3. Control Variables

We select a series of control variables that affect urban GEE from the two aspects of urban development and government factors to better study the impact of the human capital on urban green economy development. First, the urban factors include level of economic development (*LED*), city scale (*CS*), and foreign direct investment (*FDI*). Second, the government factors include Free Trade Zone (FTZ), government intervention (*GI*) and fiscal decentralization (*FD*). The specific meaning of each variable is provided below.

(1) Free Trade Zone (FTZ)

Free trade zone, China’s special functional area enjoying opening to the outside world, has greatly impacted the GTFP of China’s manufacturing industry (Liu et al., 2019) [106]. This paper uses virtual variables to measure whether a city is a free trade zone (FTZ). The variable is equal to one if the city is a free trade zone; otherwise, it is zero.

(2) Level of Economic Development (*LED*)

The LED of city is the basis for a city to achieve green growth. According to the research findings, the scale of production and consumption changes with an increase in income level, and this affects energy consumption and environmental quality [107,108]. In this study, a city’s economic development level is expressed by the logarithm of the ratio of the urban GDP value to the total population at the end of the year, i.e., the logarithm of per capita GDP.

(3) Government Intervention (*GI*)

Droste et al. [109] state that *GI* is key to urban green development. Some studies on *GI* and green economy have shown that *GI* can improve environmental performance [110] and affect the efficiency of urban pollutant emission [111]. In this article, *GI* is measured as the ratio of a city’s public budget expenditure to *GDP*.

(4) City Size (*CS*)

Theoretically, a city with a larger population has more capital for green economy development. Islam and Ghani [112] believe that population size is a key factor affecting the environment. In this article, the city scale is measured by the logarithm of the total population of each city at the end of the year.

(5) Foreign Direct Investment (*FDI*)

Foreign direct investment is an inseparable and important factor affecting China’s green economic development efficiency [113]. This factor is measured by the ratio of the total amount of foreign capital used by each city to the regional GDP.

(6) Fiscal Decentralization (*FD*)

Fiscal decentralization has a certain impact on carbon emissions, enterprise ecological innovation, and *GEE* [114,115]. This study uses fiscal autonomy to represent fiscal decentralization, i.e., the ratio of the fiscal revenue in the municipal budget to the fiscal expenditure in the municipal budget.

### 3.2. Research Methods and Model Resign

#### 3.2.1. Combines Econometric Model and LightGBM Machine Learning Algorithm

This paper empirically tests the U-shaped relationship between human capital and green growth, the transmission channel, and the contribution weight of human capital on green growth by combining econometric model and ML algorithm. The econometric model includes the benchmark model and the intermediary effect model, which can explain the direction and transmission mechanism between variables. However, it is impossible to measure the contribution of the core explanatory variable to the explained variable, and there may be some potential problems, such as the inverse causality between the independent variable and the dependent variable, or the multicollinearity between the independent variable and control variables; the machine learning algorithm can well overcome the endogenous problems and multicollinearity problems that may exist in econometric models, predict the dependent variables according to multiple explanatory variables, and accurately measure the interpretation degree of the core explanatory variables to the dependent variables, but it is difficult to explain the mechanism of the independent variables and the dependent variables. Therefore, combining the two methods can give full play to their advantages and clarify the relationship between human capital and green growth and its importance to green growth.

#### 3.2.2. Benchmark Model and Intermediary Effect Model

First, the benchmark model of human capital and green growth is as shown in Formula (1):

(1) $$GEE_{it}=\alpha_{it}+\beta HC_{it}+\gamma_{1}HC_{{}_{it}}^{2}+\sigma X_{it}+Year_{i}+City_{t}+\varepsilon_{it}$$

where *GEE_it_* is the green economic efficiency of city *i* in *t* year, *HC_it_* is the human capital of city *i* in *t* year, and *X_it_* is the control variable, mainly including *FTA*, *LED*, *GI*, *CS*, *FDI* and *FD*. *β* and *γ*_1_ are used to investigate whether there is a nonlinear relationship between *HC_it_* and *GEE_it_*. When *β* > 0 and *γ*_1_ < 0, it means that there is an inverted U-shaped relationship between *GEE* and *HC*; when *β* < 0 and *γ*_1_ > 0, there is a U-shape relationship between *GEE* and *HC*. After the regression coefficient is determined, it needs to be further determined in combination with the U test results to determine whether it is a U-shaped or inverted U-shaped relationship. *Ɛ_it_* is the residual. Year and city refer to control year and city effect, respectively.

Secondly, the intermediary effect model of human capital and green economic efficiency is as follows.

(2) $$GIN_{it}=\alpha_{it}+\beta_{1}HC_{it}++\sigma X_{it}+\varepsilon_{it}$$

(3) $$GEE_{it}=\alpha_{it}+\beta_{2}GIN_{it}+\gamma_{2}GIN_{{}_{it}}^{2}+\sigma X_{it}+\varepsilon_{it}$$

where *GIN_it_* is the green innovation of city *i* in *t* year, *HC_it_* is the human capital of city *i* in *t* year, *GEE_it_* is the green economic efficiency of city *i* in *t* year, which is the measure of green growth, and *X_it_* is the control variable, including *FTA*, *LED*, *GI*, *CS*, *FDI* and *FD*. The regression coefficient *β*_1_ reflects the relationship between *HC* and *GIN*; *β*_2_ and *γ*_2_ are used to investigate whether there is a nonlinear relationship between *GIN_it_* and *GEE_it_*. When *β*_2_ > 0 and *γ*_2_ < 0, it means that there is an inverted U-shaped relationship between *GIN* and *GEE*; when *β*_2_ < 0 and *γ*_2_ > 0, there is a U-shape relationship between *GIN* and *GEE*. After the regression coefficient is determined, it is also necessary to be in combination with U test results to determine whether it is a U-shaped or inverted U-shaped relationship. *φ_it_* is the residual. 

Then this paper takes industrial upgrading (*IU_it_*) as an intermediary variable, and uses *IU_it_* to replace *GIN_it_* in the above Equations (2) and (3), that is, to test the intermediary effect of industrial upgrading.

#### 3.2.3. LightGBM Algorithm 

When considering other factors affecting green growth, we further used the LightGBM algorithm to measure the contribution of human capital to green growth. The processing of the LightGBM algorithm is according to Fan and Liu [116]. LightGBM is an efficient implementation of XGBoost. The commonly used GBDT machine learning algorithm has limitations when processing massive data. The main reason for the birth of LightGBM is to solve the problems encountered by GBDT in massive data, so that GBDT can be better and faster used in industrial practice. Its idea is to discretize continuous floating-point features into *k* discrete values and construct a histogram with a width of *k*. Then, traverse the training data and calculate the cumulative statistics of each discrete value in the histogram. In the feature selection, we only need to traverse to find the optimal segmentation point according to the discrete value of histogram. In addition, the use of leaf wire strategy with a depth limit saves a lot of time and space consumption. Its features are: optimizing speed and memory usage; sparse optimization; optimizing accuracy; using leaf-wise growth mode, to process categorical variables; and optimizing network communication. We build a machine learning model with the help of python software. The ratio of data training set to test set is 8:2. See Appendix B for the specific hyperparametric settings of the model.

### 3.3. Data Source

This study takes the panel data of China’s 281 prefecture-level cities from 2011 to 2019 as the sample to empirically measure human capital’s impact on green growth and its internal mechanism. The data are obtained from the China Economy Database (CEIC), China City Statistical Yearbook, China Population and Employment Statistics Yearbook, and China Statistical Yearbook. When measuring the *GEE*, we obtain the data of the capital, labour, energy consumption, and GDP from CEIC; data of the SD from the China City Statistical Yearbook; and data of the two pollutants of WW and WG from CEIC. The data of human capital are obtained from China Population and Employment Statistics Yearbook and China Statistical Yearbook. The data of the intermediate variable and control variables are obtained from China City Statistical Yearbook.

Table 2 shows the sample descriptive statistics of each variable, including sample size, mean, standard deviation, minimum, maximum, Skewness, Kurtosis. The mean value of GEE is 0.334, the maximum value is 1, and the minimum value is 0.11. That is, the overall GEE is low and there is obvious regional imbalance. The difference in the HC of the different cities is relatively large. The maximum value is 16.2456, the minimum value is only 9.9059 and the mean is 13.1288. All variables are right biased except the *CS* and *IU*. In addition to *LED*, *FD* and *GIN*, the kurtosis of other variables is greater than 3, which does not obey the standard normal distribution and shows obvious characteristics of “fat-tail distribution”. The variance inflation factor (VIF) of all explanatory variables is less than 10, which means that there is no serious multicollinearity.

## 4. Empirical Results

### 4.1. Spatiotemporal Characteristics of GEE

**Figure 1:**
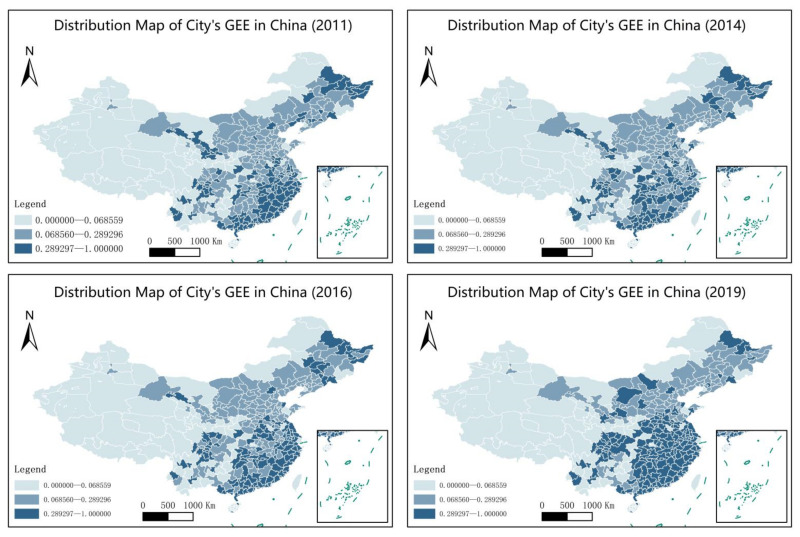
Geographical distribution map of city’s GEE in China (2011–2019).

**Figure 2:**
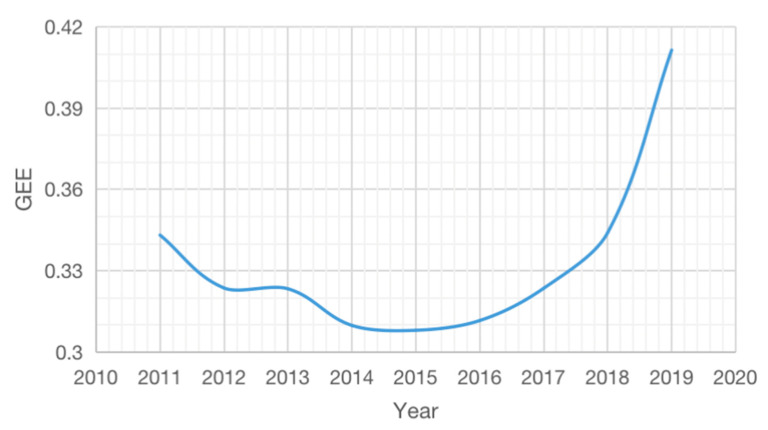
The average value change trend of GEE from 2011 to 2019.

We reveal the spatiotemporal characteristics of Chinese cities’ *GEE* and describe it using a geographic distribution map before empirically analysing the relationship between human capital and *GEE*. Chinese cities’ geographic distribution map of *GEE* (Figure 1 and Figure 2) indicates that the overall level of *GEE* is not high, and *GEE* in most cities is between 0 and 0.3341. The development level of GEE in different regions is uneven. The *GEE* level in the eastern is higher than that in the central and western regions, and the *GEE* of cities in the northeast regions has not been continuously optimized after the phased improvement. Specifically, from 2011 to 2016, some cities in the northeast regions became national new industrialization comprehensive reform pilot areas, with high overall *GEE*. However, Liaoning Province is dominated by heavy industry with high energy consumption and pollution. This industrial structure is not conducive to the continuous improvement of *GEE*. In 2019, the overall *GEE* in the northeast region decreased. Among them, the areas with the fastest improvement in *GEE* are the Yangtze River Delta and the eastern coastal areas of the Pearl River Delta, which is mainly related to the national green planning for rapid urban development during the 12th and 13th Five-Year Plans. 

**Figure 3:**
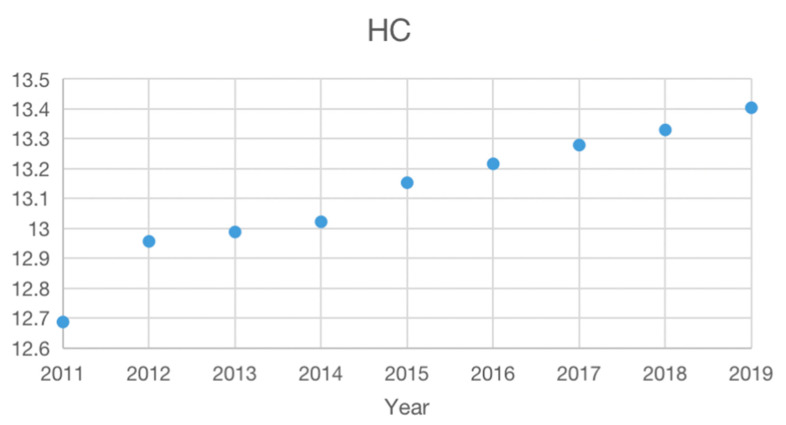
The change trend of HC from 2011 to 2019.

The average value change trend of Chinese cities’ *GEE* and *HC* from 2011 to 2019 (Figure 2 and Figure 3) indicates that Chinese cities’ *GEE* generally shows a U-shaped change, and HC is approximately linear. From the change trend of both, it is likely that HC and GEE have a U-shaped relationship. Moreover, *GEE* has been significantly improved since the 13th Five-Year Plan. This indicates that the improvement of GEE is related to government policy guidance.

### 4.2. Test of Nonlinear Relationship between HC and GG

To explore the nonlinear relationship between *HC* and *GG*, under the control of other variables, first, we use the OLS model to regress *HC* and *GEE*, and then examine with the U test. The regression results of *HC* and *GEE* are shown in Table 3. The results are as follows:

First, there is a U-shaped relationship between *HC* and *GEE*. Specifically, the regression coefficient of *HC* is −0.932, while the regression coefficient of HC^2^ is 0.037, both of which are significant at the level of 1%, indicating a U-shaped relationship between *HC* and *GEE*. On the left side of the U-shape, with the improvement of *HC*, the green growth is suppressed; when the level of *HC* exceeds a certain threshold, it will promote green growth. The results of U test show that there is a U-shaped relationship between *HC* and *GEE* at the significance level of 1%.

**Figure 4:**
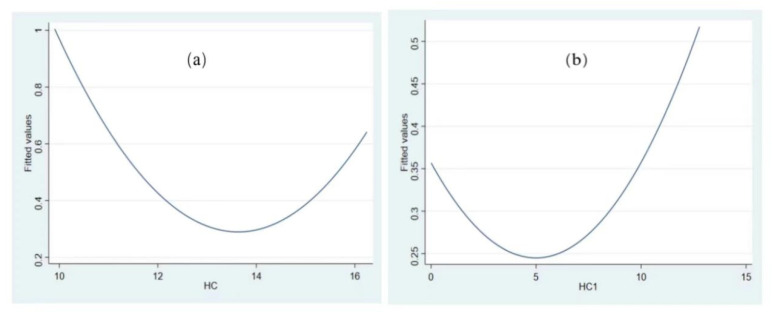
Fitting diagram of U-shaped relationship between HC and GEE, (**a**) fitting diagram of HC and GEE (HC = education expenditure) (**b**) fitting diagram of HC_1_ and GEE (HC_1_ = years of education).

Second, the current *HC* level is on the left side of the U-shape, which has not reached the threshold of *HC* promoting *GEE*. The current *HC* level is 12.469, which has not reached the threshold value of *HC* promoting *GEE* development (12.595). It is on the left side of the U-shaped fitting diagram of *HC* and *GEE* (Figure 4a).

The result that HC and green growth have a U-shaped relationship in this study supports the view of Maranzano et al. [37] to a certain extent, but the green growth measurement index is different. We adopt the NDDF-DEA model to measure *GEE* more comprehensively, while Maranzano et al. [37] adopt a carbon emission index. The result is different from Wang et al. [22] and Xiao and You [88]. They all support that total *HC* can improve green growth, and Wang et al. [22] further conclude that different HC levels have different effects on GTFP.

**Table 3:** Regression results of U-shaped relationship between HC and GEE.

Variables	GEE
*HC*	−0.932 ***
	(−7.71)
*HC^2^*	0.0370 ***
	(8.03)
*FTA*	0.0010
	(0.14)
*LED*	0.0260
	(1.57)
*GI*	−0.8730 ***
	(−3.67)
*CS*	0.1150 ***
	(2.85)
*FDI*	0.1610
	(0.14)
*FD*	0.1280 ***
	(2.72)
U test	12.469 ***
	(5.95)
U test lower bound interval	9.9060
U test upper bound interval	16.2460
_cons	4.892 ***
	(5.57)
Year	controlled
City	controlled
N	2493
R^2^	0.7640

Note: (1) *t* statistics in parentheses; (2) *** represent significance levels of 1.

### 4.3. The Mechanism Test Results Analysis

#### 4.3.1. Human Capital, Green Innovation, and Green Growth

We use the intermediary effect model to examine whether green innovation acts as an intermediary variable between *HC* and *GEE*. The empirical results are shown in Table 4.

The results in column (1) of Table 4 shows that there is a U-shaped relationship between *HC* and *GEE*; the results in column (2) show that *HC* significantly and positively promotes the development of green innovation at the significance level of 1%, and the regression coefficient is 0.332, that is, every 1% increase in *HC* increases green innovation by 0.332%. The results in column (3) shows that the regression coefficient of *GIN* is significant at the level of 1%, which is −0.051, while the coefficient of *GIN^2^* is positive at the significance level of 1%. According to *GIN* and *GIN^2^* coefficients, there may be a U-shaped relationship between green innovation and *GEE*. Before the green innovation level reaches the threshold value, green innovation suppresses *GEE*. Once the green innovation level reaches the threshold value, the high utilization rate of resources promotes the development of *GEE*. The U-shaped relationship between green innovation and *GEE* supports the views of Hu et al. [63] and Liu et al. [117]. The U test results also show that the U-shaped relationship between green innovation and *GEE* is significant at the level of 1%. This means that human capital is positively promoting green innovation, and there is a U-shaped relationship between green innovation and *GEE*. This verifies hypothesis 2 that green innovation acts as transmission channel between *HC* and *GEE*.

#### 4.3.2. Human Capital, Industrial Upgrading and Green Growth

We use the intermediary effect model to test whether industrial upgrading acts as an intermediary variable between *HC* and *GEE*. The empirical results are shown in Table 5.

The results in column (2) of Table 5 show that *HC* significantly promotes industrial upgrading. The regression coefficient is 0.039, which means that every 1% increase in *HC* will improve industrial upgrading by 0.039%; the results in column (3) of Table 5 show that the regression coefficient of *IU* is negative and that of *IU*^2^ is positive, indicating that there is a U-shaped relationship between industrial upgrading and *GEE*. On the left side of the U-shaped turning point, that is, the “accumulation” stage of industrial upgrading, with the development of industrial upgrading, industrial upgrading inhibits *GEE*; once the industrial upgrading exceeds the threshold and enters the “leap” stage of industrial upgrading, the industrial upgrading is dominated by the development of high-tech and digital industries, which improves the utilization rate of resources and promotes *GEE*. The U test results further verify that the U-shaped relationship between industrial upgrading and *GEE* is significant. On the whole, *HC* is positively promoting industrial upgrading. There is a U-shaped relationship between industrial upgrading and GEE. This verifies hypothesis 3 that industrial upgrading is the transmission channel between *HC* and *GEE*.

### 4.4. Robustness Test

To prove that the conclusion is reliable, we examine the robustness of the benchmark model. The robustness test can be carried out by replacing either dependent variables or independent variables.

#### 4.4.1. Using Substitute Variables of Human Capital

To further test the U-shaped relationship between human capital and green growth, we use the human capital measured by education years in each prefecture-level city [118,119] to replace current human capital measured by education expenditure to verify the relationship between human capital and green growth. The regression results in Column (1) of Table 6 and Figure 4b show that the relationship between human capital and *GEE* is U-shaped and has passed the U test. On the left side of the U-shaped turning point, when human capital increases, it inhibits green growth; when human capital level exceeds a certain threshold, it promotes green growth.

#### 4.4.2. Using Substitute Variables of GEE

We use *CO*_2_ emissions to replace the dependent variable, i.e., *GEE*, and verify the relationship between human capital and green growth through the relationship between human capital and *CO*_2_ emissions. The regression results in column (2) of Table 6 reveal that the coefficients of *HC* and *HC*^2^ are 1.294 and −0.046, respectively, and are significant at the level of 1%, indicating that the relationship between human capital and *CO*_2_ emissions is an inverted U-shape and has passed the U test. Since lower *CO*_2_ emissions mean higher *GEE*, this demonstrates the U-shaped relationship between human capital and *GEE*. On the left side of the U-shape, *CO*_2_ emissions are increasing as human capital enhances, indicating that human capital inhibits green growth. When human capital level exceeds a certain threshold, an increase in human capital will reduce *CO_2_* emissions, thus promoting green growth.

### 4.5. Heterogeneity Analysis

#### 4.5.1. Heterogeneity Analysis Based on Different Location of Cities

**Figure 5:**
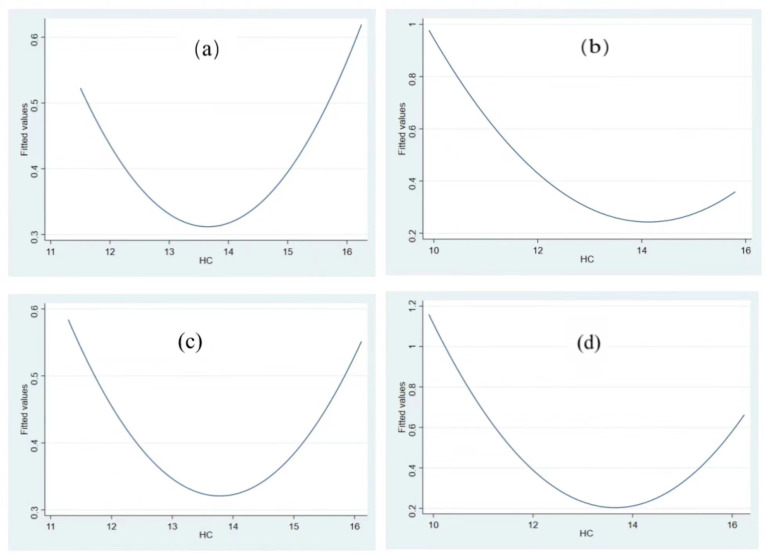
Fitting diagram of city’s HC and GEE in different locations. Note: (**a**) fitting diagram of HC and GEE for eastern cities; (**b**) fitting diagram of HC and GEE for western cities; (**c**) fitting diagram of HC and GEE for southern cities; (**d**) fitting diagram of HC and GEE for northern cities.

Since human capital development level differs in different regions, its relationship with green growth may also be different. According to the location of cities, we divide the samples into eastern, central, and western regions, and examine the relationship between human capital and *GEE* in different regions. The results are shown in Table 7 and Figure 5. The regression results indicate that the relationship between human capital and *GEE* in eastern, central, and western regions is U-shaped. The U test results in the eastern and western regions are significant, but insignificant in the central region. We draw a conclusion that eastern cities’ human capital level (13.635) is higher than that of central cities (12.9394); and that in central cities is higher than that of western cities (12.457). Both the regression results and U-test demonstrates that there is a significant U-shaped relationship between *HC* and *GEE* in eastern and western cities, but not in central cities. Both eastern and western cities’ human capital level is on the left side of the threshold (Figure 5). The development of human capital still inhibits *GEE*. Compared with the western region, the eastern region is closer to the U-shaped threshold.

Moreover, we further divide the samples into southern and northern regions, and examines the relationship between human capital and *GEE* in different regions. The results are shown in Table 7 and Figure 5. Both the regression and the U test results indicate that there is a significant U-shaped relationship between human capital and *GEE* in the southern and northern regions.

Specifically, the human capital level in southern cities (14.176) is higher than that in northern cities (12.066). The southern cities’ human capital level is already on the right side of the threshold (14.136), indicating that it will promote *GEE* development as it improves. Human capital level in northern cities is 12.066, which is still on the left side of the threshold for northern cities (12.12, Figure 5d). That means human capital in northern cities still inhibits *GEE*. Southern cities’ human capital promotes *GEE*, while northern cities’ human capital inhibits *GEE*. This result is also consistent with the fact that human capital and city development levels in the southern cities are higher than those in the northern cities.

#### 4.5.2. Heterogeneity Analysis of Different Size of Cities

**Figure 6:**
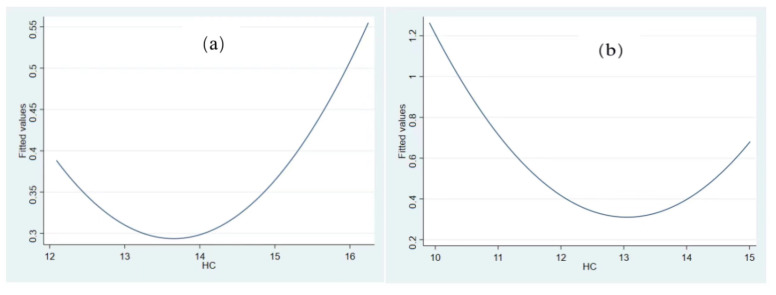
Fitting diagram of HC and GEE for different city size. (**a**) Fitting diagram of HC and GEE in large cities; (**b**) fitting diagram of HC and GEE in small and medium cities.

Influenced by resource endowment, cities of different size have different human capital development level. According to cities’ development size, we divided the samples into large cities and small and medium-sized cities for heterogeneity analysis. If the urban population in that year is larger than the sample average level, it is considered as a big city; otherwise, it is regarded as a small and medium-sized city. The regression and U test results reveal that (Table 8) the human capital level in large cities (13.665) is higher than that in small and medium-sized cities (11.556). There is a significant U-shaped relationship between *HC* and *GEE* in both large and small and medium-sized cities. The human capital level in large cities (13.665) is on the right side of the threshold (13.558) (Figure 6a). The relationship between *HC* and *GEE* exceeds the U-shaped turning point, indicating that *HC* will promote *GEE*. Small and medium-sized cities’ human capital level (11.556) is still on the left side of the threshold (11.591) (Figure 6b), which has not yet reached the U-shaped threshold. It is still in the state of *HC* inhibiting *GEE*, which means that city scale development level will speed up crossing the threshold between *HC* and *GEE*, helping *HC* promote *GEE*.

### 4.6. Contribution of HC to GEE

**Figure 7:**
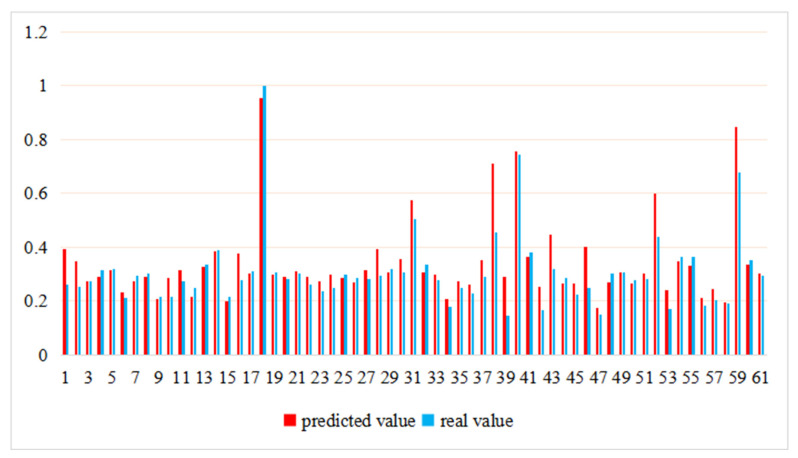
The comparison of GEE predicted value and real value.

Given other factors affecting the *GEE*, we further use LightGBM to measure the contribution of *HC* to *GEE*. Based on the six indices (*HC*, *FTZ*, *LED, GI*, *CS*, *FDI* and *FD*), this study uses the LightGBM machine learning method to predict urban *GEE* and fit it with the actual *GEE*. The fitting result is shown in Figure 7 and Table 9. Figure 7 shows that the general trend of the predicted value and actual values is the same. Further, the prediction performance results of LightGBM presented in Table 9 indicate that the R-squared value (R^2^) of the training set is 0.886, and the R^2^ value of the test set is 0.695, which implies that *HC* and the selected control variables are the main factors affecting the *GEE*.

**Figure 8:**
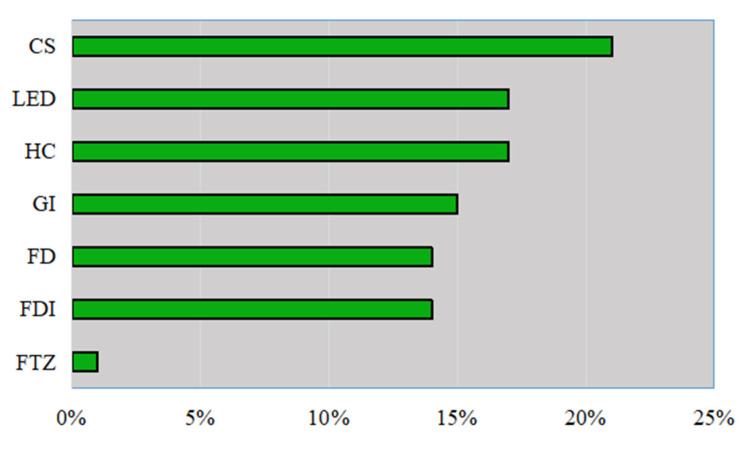
The contribution for determinants of GEE.

An analysis of the relative importance of each variable to the *GEE* (Figure 8) reveals that the contribution of *CS* is the highest, reaching 21%. The contribution of *HC* and *LED* are 17%, respectively, second only to *CS*. The contributions of *GI, FDI* and *FD* are 15%, 14%, and 14%, respectively. The contribution of *FTZ* is the smallest, only 1%. Based on the contribution of various independent variable and control variables, we find that *CS*, *LED* and *HC* are the three main factors affecting *GEE*. *HC* is the second largest factor affecting *GEE*, second only to *CS*. *CS* and *HC* reflect the quantity and quality of urban population, and their total contribution is 38%. This is because people are the intrinsic factors that affect *GEE*; the other control variables include the extrinsic factors that affect *GEE*.

## 5. Conclusions and Policy Implications

### 5.1. Conclusions

With the rapid industrialization and urbanization, the increasing imbalance between economic system and ecosystem causes serious problems such as global warming, extreme climate, and frequent natural disasters, posing a great threat to human society. Therefore, the key path for countries around the world toward sustainable development is to transform to a “green growth” model that takes into account both economic growth and environmental protection. Meanwhile, China’s human capital level has been continuously improving since the reform and opening up. Naturally, then, it raises a question for academics and policy authorities: what is the relationship between human capital and green growth? To answer this question, this paper selects the sample city data of China’s 281 prefecture-level cities (including municipalities directly under the Central Government) and analyses the question in great detail from a theoretical perspective and at an empirical level. First, by reviewing classical literature, we put forward the hypothesis of U-shaped relationship between human capital and green growth. Then, we introduce the NDDF-DEA model to measure China’s sample cities’ green growth level during the statistical period. On this basis, we empirically test the previous research hypotheses by using econometric model, and measure the contribution of human capital to green growth by ML algorithm. 

This paper has the following main findings: (1) China’s human capital and green growth have a U-shaped relationship. Before reaching a certain threshold, human capital will inhibit green growth. After exceeding a certain threshold, human capital will promote green growth. Green innovation and industrial upgrading are transmission channels when human capital impacts green growth. (2) When considering other factors influencing green growth, human capital is very important. HC and economic growth have the same contribution weight to GG (17%), ranking two, second only to city size (21%). (3) The influence of human capital on green growth in China is characterized by regional imbalance and urban scale imbalance. It is good to hear that in the southern regions, human capital has surpassed the “U-shaped” threshold, promoting green growth. In contrast, however, human capital in northern China negatively impacts green growth. There is a significant U-shaped relationship between human capital and green growth in the eastern and western regions while the U-shaped relationship between the two in the central region is not significant. In the eastern regions, the current level of human capital is closer to the U-shaped inflection point. That is, when human capital level continues to improve, It will soon have a positive impact on green growth. But the human capital level in the western region still cannot reach the U-shaped threshold, which currently inhibits green growth. From the perspective of urban scale, the human capital of large cities has exceeded the U-shaped inflection point and played a role in promoting green growth; the human capital of small and medium-sized cities is still far from the U-shaped inflection point, which has a restraining effect on green growth. The level of urban scale development will accelerate the threshold crossing between HC and GG, and promote the positive correlation effect of HC on GG.

### 5.2. Policy Implications

Based on these findings, we provide the following relevant policy implications:

(1) Developing economies should pay full attention to the important value of education investment and talent cultivation in green transformation. Decision makers should regularly and dynamically assess human capital stock, accurately estimate human capital, and classify different talent development levels in various regions. They then can formulate matching talent development strategies and industrial policies to help improve human capital development levels and promote green growth.
(2) Companies (especially environmentally sensitive companies) should work hard to shape a corporate culture centred on knowledge management, green innovation, and people-orientation. They should spare no effort to build a talent echelon, greatly enhance the training of employees’ skills, give full play to talents’ subjective initiative, motivate employees’ innovative practices, and realize the marginal incremental effect of human capital on companies’ GTFP, promoting the transformation of green innovation achievements and industrial upgrading.
(3) Urban governance authorities in northern, central and western regions and small and medium-sized cities should rationally recognize the current shortcomings of HC development. On the one hand, they should increase the ratio of education expenditure in public expenditure, and gradually improve local population’s education and skill level, so as to promote human capital development to a high level. On the other hand, given the location characteristics and resource endowments, they should actively explore the talent introduction policy for sustainable development and improve the supporting software and hardware infrastructure to attract top talents and value conversion. By adopting these measures, they can gradually use top talents to promote local green growth so as to narrow the regional gap of green growth with the eastern, southern regions and large cities.

### 5.3. Limitations of This Paper

The deficiency of the paper is that it fails to further distinguish human capital into academic education and skill education. In the future, we can do more detailed research on the impact of different types of education and different levels of HC on GG.

## Figures and Tables

**Table 1:** Variable definition and calculation method.

Variable Type	Definition	Code	Calculation Method
Dependent variable	Green economic efficiency	*GEE*	Measured by the NDDF-DEA model
Independent variable	Human capital	*HC*	Logarithm of financial education expenditures in prefecture-level cities at the end of each year
Intermediary variable	Green innovation	*GIN*	Logarithm of green invention patent applications in prefecture-level cities
	Industrial upgrading	*IU*	The output value of secondary industry plus output value of tertiary industry, divided by GDP
Control variable	Free trade zone	*FTA*	The variable is equal to one if the city is a free trade zone; otherwise, it is zero
	Level of economic development	*LED*	Logarithm of per capita GDP
	Government intervention	*GI*	Public budget expenditure divided by GDP
	City scale	*CS*	Logarithm of the total population of each city at the end of the year
	Foreign direct investment	*FDI*	The total amount of foreign capital divided by GDP
	Fiscal decentralization	*FD*	The ratio of the fiscal revenue in the municipal budget to the fiscal expenditure in the municipal budget
Other variables	Years of education	*HC*_1_	(number of university students in city/number of university students in province) × ln(6 × the proportion of labour force in the sample with no higher than primary school education +9* the proportion of labour force with no higher than junior middle school education +12 × the proportion of labour force with no higher than senior high school education +16* the proportion of labour force with college education) (Wang et al. 2021) [22]
	Carbon dioxide emissions	*CO_2_*	Logarithm of carbon dioxide emissions
	Year	*Year*	a dummy variable
	City	*City*	a dummy variable according to China Urban Statistical Yearbook

**Table 2:** The statistics summary of variables.

Variable	Obs	Mean	Std. Dev.	Min	Max	Skewness	Kurtosis	VIF
*GEE*	2297	0.3341	0.1623	0.1107	1.0000	2.6018	10.8540	
*HC*	2297	13.1288	0.7805	9.9059	16.2456	2.4250	9.0127	8.6100
*FTA*	2297	0.2037	0.4029	0	1.0000	1.5530	3.4119	7.8900
*LED*	2297	10.7171	0.5790	8.8416	13.0557	0.2192	2.8621	5.5300
*GI*	2297	0.0793	0.0281	0.0234	0.2273	1.1885	5.6820	4.5200
*CS*	2297	5.9025	0.6963	2.9704	8.1362	−0.5567	4.0910	2.3700
*FDI*	2297	0.0027	0.0027	0	0.0299	2.2460	13.5210	1.3500
*FD*	2297	0.4790	0.2255	0.0680	1.5413	0.5302	2.6254	1.2400
*G* *IN*	2297	4.3325	1.7641	0	10.1825	0.4849	2.9046	4.2300
*IU*	2297	4.4730	0.1035	3.6618	4.6049	−2.1419	11.1377	2.0000

**Table 4:** Empirical results of the relationship between HC, green innovation and GEE.

Variables	(1)	(2)	(3)
	*GEE*	*GIN*	*GEE*
*HC*	−0.932 ***	0.332 ***	
	(−7.71)	(3.94)	
*HC* ^2^	0.037 ***		
	(8.03)		
*GIN*			−0.051 ***
			(−6.97)
*GIN* ^2^			0.008 ***
			(10.17)
*FTA*	0.001	−0.021	0.001
	(0.14)	(−0.63)	(0.21)
*LED*	0.026	0.467 ***	0.034 **
	(1.57)	(5.67)	(2.27)
*GI*	−0.873 ***	2.168 *	−0.788 ***
	(−3.67)	(1.82)	(−3.46)
*CS*	0.115 ***	0.576 ***	0.097 **
	(2.85)	(2.87)	(2.56)
*FDI*	0.161	−1.049	0.805
	(0.14)	(−0.18)	(0.70)
*FD*	0.128 ***	−0.134	0.123 ***
	(2.72)	(−0.57)	(2.66)
U test	12.469 ***		3.073 ***
	(5.95)		(6.97)
U test lower bound interval	9.906		0
U test upper bound interval	16.246		10.182
_cons	4.892 ***	−6.294 ***	−0.726 **
	(5.57)	(−3.88)	(−2.26)
N	2493.000	2493.000	2493.000
R^2^	0.764	0.950	0.768

Note: (1) t statistics in parentheses; (2) * *p* < 0.1, ** *p* < 0.05, *** *p* < 0.01

**Table 5:** Empirical results of the relationship between HC, industrial upgrading and GEE.

	(1)	(2)	(3)
	*GEE*	*IU*	*GEE*
*HC*	−0.932 ***	0.039 ***	
	(−7.71)	(4.65)	
*HC^2^*	0.037 ***		
	(8.03)		
*IU*			−5.770 ***
			(−3.90)
*IU^2^*			0.708 ***
			(4.01)
*FTA*	0.001	0.008 **	0.002
	(0.14)	(2.42)	(0.37)
*LED*	0.026	0.059 ***	0.033 ***
	(1.57)	(7.19)	(1.98)
*GI*	−0.873 ***	−0.228 *	−0.478 **
	(−3.67)	(−1.92)	(−2.02)
*CS*	0.115 ***	−0.065 ***	0.216 ***
	(2.85)	(−3.27)	(5.70)
*FDI*	0.161	0.012	−0.598
	(0.14)	(0.02)	(−0.46)
*FD*	0.128 ***	0.119 ***	0.042
	(2.72)	(5.04)	(0.87)
U test	12.469 ***		4.074 **
	(5.95)		(3.07)
U test lower bound interval	9.906		3.66
U test upper bound interval	16.246		4.60
_cons	4.892 ***	3.702 ***	10.279 **
	(5.57)	(22.90)	(3.28)
N	2493.000	2417.000	2417.000
R^2^	0.764	0.862	0.757

Note: (1) t statistics in parentheses; (2) * *p* < 0.1, ** *p* < 0.05, *** *p* < 0.01.

**Table 6:** Robustness test of the U-shaped relationship between human capital and *GEE*.

Variables	*GEE*	*CO_2_*
	(1)	(2)
*HC_1_*	−0.028	1.294 ***
	(−1.25)	(3.39)
*HC*_1_^2^	0.007 ***	−0.046 ***
	(3.29)	(−3.31)
*FTZ*	0.005	0.038 *
	(0.76)	(1.86)
*LED*	0.065 ***	0.192 ***
	(4.09)	(3.66)
*GI*	−0.543 **	−0.035
	(−2.24)	(−0.05)
*CS*	0.207 ***	0.162
	(5.33)	(1.27)
*FDI*	−0.207	−10.045 ***
	(−0.16)	(−2.74)
*FD*	0.074	−0.105
	(1.52)	(−0.71)
U test	2.018 *	14.051 **
	(1.25)	(1.80)
U test lower bound interval	0	9.906
U test upper bound interval	12.782	16.256
_cons	−2.334 ***	−2.852
	(−5.93)	(−1.03)
N	2297.000	2482.000
R^2^	0.758	0.952

Note: (1) t statistics in parentheses; (2) ***, ** and * represent significance levels of 1%, 5% and 10%, respectively; (3) column (1) is the regression result of *HC_1_* and *GEE*; column (2) is the regression result of *HC* and *CO_2_* emissions.

**Table 7:** Test of the relationship between HC and GEE in different regions.

Variables	East	Centre	West	South	North
	(1)	(2)	(3)	(4)	(5)
	*GEE*	*GEE*	*GEE*	*GEE*	*GEE*
*HC*	−0.749 ***	−0.609 **	−1.026 ***	−0.933 ***	−1.115 ***
	(−4.37)	(−2.18)	(−4.06)	(−6.44)	(−4.73)
*HC^2^*	0.027 ***	0.030 ***	0.041 ***	0.033 ***	0.046 ***
	(4.23)	(2.75)	(4.13)	(6.07)	(4.95)
*FTZ*	−0.001	−0.026 **	0.048 ***	−0.011	0.007
	(−0.11)	(−1.98)	(3.24)	(−1.47)	(0.55)
*LED*	0.066 ***	0.078 **	−0.161 ***	0.025	−0.087 ***
	(3.28)	(2.23)	(−4.25)	(1.16)	(−2.75)
*GI*	−0.642 **	−2.309 ***	−1.491 ***	−0.500 *	−0.899 **
	(−2.01)	(−5.05)	(−2.87)	(−1.68)	(−2.11)
*CS*	0.460 ***	0.086	−0.234 **	0.145 ***	−0.089
	(5.35)	(1.54)	(−2.55)	(3.15)	(−0.94)
*FDI*	−0.189	4.157 *	0.162	−4.781 ***	3.683 *
	(−0.13)	(1.77)	(0.04)	(−2.90)	(1.79)
*FD*	0.048	0.405 ***	0.188	0.112 **	0.087
	(0.74)	(4.68)	(1.53)	(2.00)	(0.94)
U test	13.635 **	10.238	12.457 ***	14.176 ***	12.066 ***
	(2.98)	(0.28)	(3.44)	(3.22)	(3.61)
U test lower bound interval	9.906	9.906	9.906	9.906	9.906
U test upper bound interval	16.246	16.246	16.246	16.246	16.246
_cons	1.536	1.622	9.716 ***	5.561 ***	8.399 ***
	(1.08)	(0.86)	(5.20)	(5.23)	(4.76)
N	956.000	759.000	702.000	1607.000	810.000
R^2^	0.839	0.759	0.771	0.737	0.804

Note: (1) t statistics in parentheses; (2) * *p* < 0.1, ** *p* < 0.05, *** *p* < 0.01.

**Table 8:** Test of the relationship between HC and GEE for different city scale.

Variables	Big Cities	Small and Medium−Sized Cities
	*GEE*	*G* *EE*
*HC*	−1.166 ***	−1.020 ***
	(−6.54)	(−3.55)
*HC* ^2^	0.043 ***	0.044 ***
	(6.45)	(3.80)
*FTZ*	−0.015 **	0.021 *
	(−2.15)	(1.85)
*LED*	0.113 ***	−0.041
	(4.88)	(−1.63)
*GI*	−1.354 ***	−0.624 *
	(−4.43)	(−1.75)
*CS*	−0.197 ***	0.111 *
	(−2.63)	(1.84)
*FDI*	−0.722	2.376
	(−0.57)	(1.22)
*FD*	0.213 ***	0.065
	(3.70)	(0.88)
U test	13.665 ***	11.566 ***
	(5.11)	(2.37)
U test lower bound interval	9.906	9.906
U test upper bound interval	16.256	16.256
_cons	8.490 ***	5.914 ***
	(5.94)	(3.10)
N	1323.000	1159.000
R^2^	0.732	0.789

Note: (1) *t* statistics in parentheses; (2) * *p* < 0.1, ** *p* < 0.05, *** *p* < 0.01.

**Table 9:** The performance measurement of GEE by LightGBM.

	MSE	RMSE	MAE	MAPE	R^2^
training set	0.003	0.054	0.034	9.834	0.886
test set	0.007	0.082	0.059	16.618	0.695

## Data Availability

The data used to support the findings of this study are available from the corresponding author upon request.

## Appendix A

**Table A1:** Full term and the abbreviations.

Number	Full Term	Abbreviation	Number	Full Term	Abbreviation
1	Green economic efficiency	GEE	12	Years of education	HC_1_
2	green growth	GG	13	carbon dioxide emissions	CO_2_
3	Human capital	HC	14	green total factor productivity	GTFP
4	Green innovation	GIN	15	sulfur dioxide	SO_2_
5	Industrial upgrading	IU	16	total factor productivity	TFP
6	Free trade zone	FTA	17	data envelopment analysis	DEA
7	Level of economic development	LED	18	non-radial direction distance function	NDDF
8	Government intervention	GI	19	machine learning	ML
9	City scale	CS	20	Shephard distance function	SDF
10	Foreign direct investment	FDI	21	directional distance function	DDF
11	Fiscal decentralization	FD			

## Appendix B

**Table A2:** The parameter values based on LightGBM machine learning algorithm.

Parameter	Parameter Value
Training time	0.219 s
Data segmentation	0.8
Data shuffle	Yes
Base learner	GBDT
Base learner number	130
Learning rate	0.1
L1 regular term	0
L2 regular term	1
Sample sign sampling rate	1
Tree feature sampling rate	1
Maximum depth of tree	10
Leaf node minimum sample	15
